# Analysis of antibodies from whole-cell immunization by a tANCHOR cell-based ELISA

**DOI:** 10.17912/micropub.biology.001201

**Published:** 2024-04-17

**Authors:** Hubert Bernauer, Anja Schlör, Josef Maier, Katja Hanack, Norbert Bannert, Daniel Ivanusic

**Affiliations:** 1 ATG:biosynthetics GmbH, Weberstraße 40, 79249 Merzhausen, Germany; 2 new/era/mabs GmbH, August-Bebel-Str. 89, 14482 Potsdam, Germany; 3 Institute for Biology and Biochemistry, University of Potsdam, Karl-Liebknechtstr. 24-25, 14476 Potsdam, Germany; 4 IStLS, Härlestr. 24/1, 78727 Oberndorf a.N., Germany; 5 Sexually transmitted bacterial pathogens and HIV (FG18), Robert Koch-Institute, Nordufer 20, 13353 Berlin, Germany.

## Abstract

Monitoring specific antibodies derived from whole-cell immunization through cell-based ELISA methods poses challenges due to humoral responses against various cell proteins. In this report, we outline a technique involving pre-adsorption on cells to remove undesirable antibodies from immune serum. This step provides the subsequent monitoring of antibodies specific to the targeted antigen using a tANCHOR-based ELISA. Notably, this approach accelerates result acquisition, eliminating the necessity to purify the expressed antigen or obtain a customized peptide for coating assay plates.

**Figure 1. Analysis of antibodies from whole-cell immunization:**
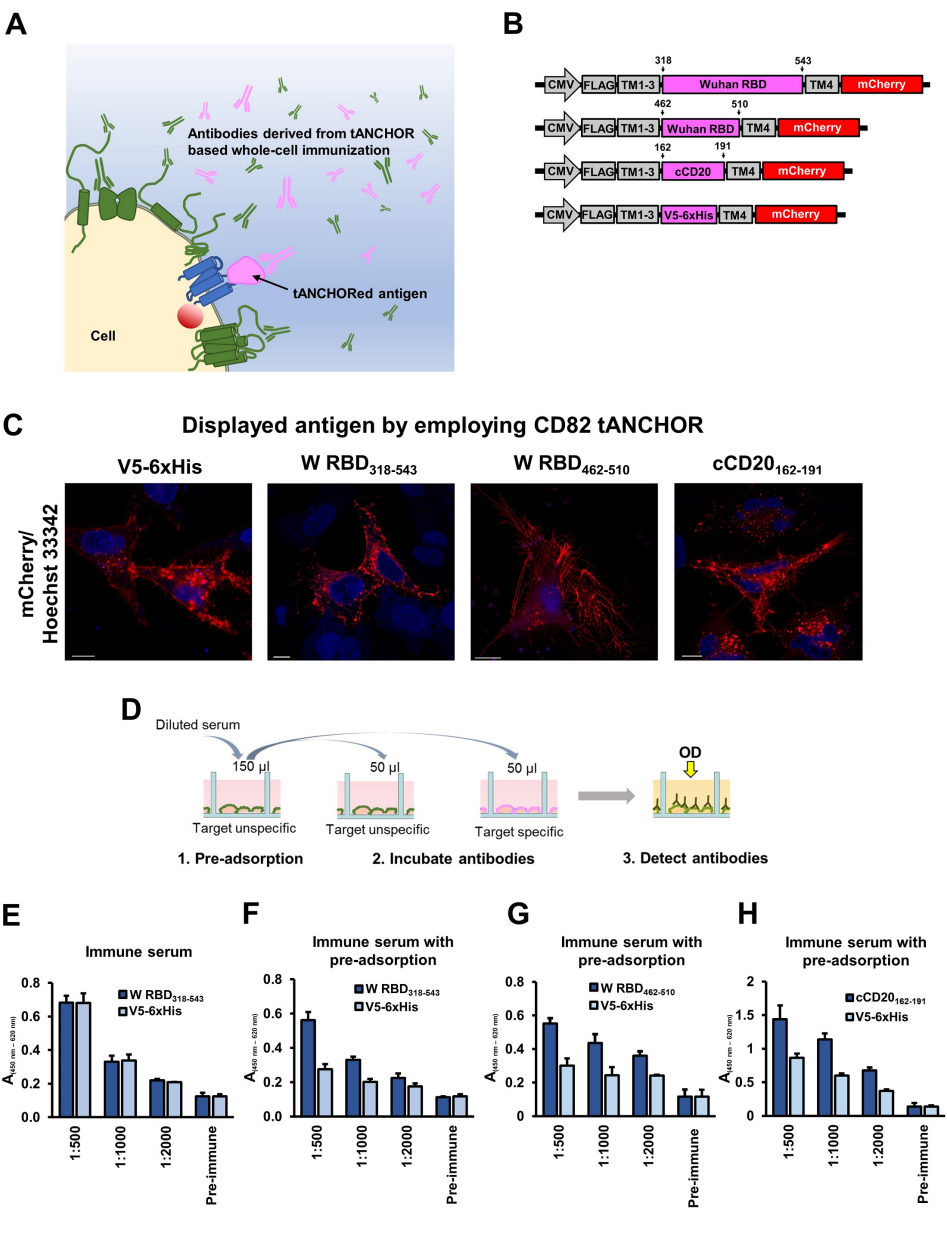
( **A** ) Cartoon illustrating the difficulty when antibodies are monitored derived from whole-cell immunization using the tANCHOR system. Antibodies are induced against target-specific (pink) and target-unspecific (green) epitopes. ( **B** ) Schematic presentation of expression constructs used in the study (not to scale). Numbers refer to amino acid positions. ( **C** ) Confocal laser scanning microscopy (CLSM) images of HeLa cells transfected with indicated tANCHOR expression constructs containing coding DNA sequences for displaying V5-6xHis background control peptide, two versions of the SARS-CoV-2 Wuhan-Hu-1 receptor-binding domain (W RBD), or the canine CD20 (cCD20) extracellular loop. Cell nuclei were stained with Hoechst 33342. Scale bars, 10 µm. ( **D** ) Illustration presenting workflow for measuring target-specific antibodies derived from whole-cell immunization. Results of cell-based ELISA of serum derived from whole-cell immunization of mice (BALB/c) with the construct ptANCHOR-CD82-RBD _318-543_ -mCherry ( **E** ) without or ( **F** ) with pre-incubation on HeLa cells transfected with pCMV-CD82-V5-6xHis-mCherry. Testing of serum derived from an immunization study immunized with HEK293T cells transfected ( **G** ) with ptANCHOR-CD82-RBD _462-510_ -mCherry or ( **H** ) ptANCHOR-CD82-cCD20 _162-191_ -mCherry.

## Description

The induction of antibodies through whole-cell immunization is a well-established method, particularly effective for eliciting immune responses against full-length proteins (Dreyer et al., 2010; Huang et al., 2019; Rezaei and Ghaderi, 2017, 2018) . Moreover, we previously demonstrated that with the tANCHOR display technology, it is feasible to induce antibodies against any protein sequences and peptides that are displayed on the cell surface (Bernauer et al., 2023) . The tANCHOR whole-cell immunization technique enables the expression of antigens that are inserted by exchanging the large extracellular loop (LEL) of a tetraspanin. The insertion of a protein sequence of interest replaces, therefore, the LEL of the tetraspanin sequence. The tetraspanin CD82, serving as an anchor for displaying antigens on the cell surface, is particularly well-suited for this purpose. (Ivanusic et al., 2021) . Humoral immune responses induced by immunization with whole cells displaying anchor-inserted epitopes can be easily detected using peptides or purified proteins containing these epitopes coated on assay plates (Bernauer et al., 2023) . If coating of assay plates with proteins is not desired or peptides with all necessary specifications (e.g., post-translational modifications like glycosylation) are not available, the utilization of a cell-based ELISA is an alternative method (Molnár, 2019; Pandey et al., 2019; Shan et al., 2016; Versteeg et al., 2000). In this approach, cells expressing tetraspanin-anchored peptides or proteins employed for immunization can serve as a basis for the antibody cell-based assay. However, when employing whole cells for antigen presentation, it is essential to acknowledge that the recipient's immune system might not only respond to the specific peptide or protein displayed by the tANCHOR system (Dreyer et al., 2010) . This becomes particularly evident when cells from a xenogeneic species are utilized for vaccination. In fact, it makes it very challenging to apply a cell-based ELISA for monitoring target-specific antibodies in a pool of irrelevant antibodies. ( **Fig. 1A** ). When analyzing serum or plasma IgG antibodies from such a vaccination through a cell-based antibody detection assay, the assay will measure the binding of all induced antibodies to various host proteins, not solely the antibodies specific to the antigen displayed by the tANCHOR system. Therefore, we thought to modify the assay in order to establish a reliable, fast, and cost-efficient cell-based ELISA technique that confines detection to antibodies directed to the protein or peptide inserted in the tANCHOR display system. This enables us to confirm successful whole-cell immunization against the target protein before proceeding to generate hybridoma cell lines for IgG production. In contrast to other reported whole-cell immunization studies, which typically utilize cell-based or cell-lysate ELISA for IgG monitoring after establishing hybridoma cell lines (Balouchi-Anaraki et al., 2021; Chen et al., 2018; Dreyer et al., 2010; Huang et al., 2019; Rezaei and Ghaderi, 2017, 2018) , our approach allows for testing directly IgG titers form immune serum. This facilitates the selection of mice with the highest target-specific IgG titer without the need for purified proteins or custom peptides. Consequently, the preselection of mice with high target-specific serum antibody titers expedites the generation of monoclonal antibodies (Holzlöhner and Hanack, 2017; Leenaars and Hendriksen, 2005; Phakham et al., 2022). To establish this ELISA method, we employed serum obtained from mice previously immunized in a study reported elsewhere (Bernauer et al., 2023) . In brief, the mice were immunized with human embryonic kidney (HEK) 293T cells that contain a surface decorated with antigens by using the tANCHOR display system. We immunized against the receptor-binding domain (Wuhan-Hu-1 RBD _318-543_ or RBD _462-510_ ) of SARS-CoV-2 and the extracellular loop of canine CD20 (cCD20 _162-191_ ) ( **Fig. 1B** ). Five weeks post-immunization, we successfully monitored the induction of IgG antibodies the targets in the obtained sera. This was achieved through the utilization of protein- and peptide-coated assay plates, as reported earlier (Bernauer et al., 2023) . Using these sera, we set out to establish the whole cell-based IgG-ELISA for specific detection and quantification of these antibodies. For this, we expressed the Wuhan-Hu-1 RBD comprising ( *i* ) amino acids (aa) 318–543, ( *ii* ) a truncated RBD aa 462-510, ( *iii* ) cCD20 _162-191_ or ( *iv* ) a non-relevant protein (V5-6xHis as a control) inserted into the tetraspanine CD82 (tANCHOR) on the surface of HeLa cells ( **Fig. 1C** ). In order to focus the detection specifically on antibodies against the relevant RBD or CD20 peptides, we opted to pre-adsorb the sera with HeLa cells displaying the V5-6xHis peptide. This additional step was implemented to largely eliminate antibodies binding to other cellular epitopes from the diluted sera. The sera, pre-adsorbed in this manner, were subsequently employed in a standard whole-cell ELISA, utilizing cells displaying the RBD or CD20 peptides ( **Fig. 1D** ). To validate this approach, we generated 1:500, 1:1,000, and 1:2,000 dilutions of sera from immunized mice. Subsequently, these sera dilutions were pre-adsorbed on cells displaying the V5-6xHis peptide. After the pre-adsorption process, the diluted serum was evaluated for its binding affinity to HeLa cells presenting target-specific epitopes, while the V5-6xHis peptide was utilized as a control. To demonstrate the effect of pre-adsorption we tested in parallel the same serum without pre-adsorption using cells displaying the RBD _318-543_ protein or the V5-6xHis peptide. Without pre-adsorption step the absorbance values obtained with cells expressing the RBD _318-543_ or the V5-6xHis peptide are comparable ( **Fig. 1E** ). However, pre-adsorption of the serum led to a significantly lower absorbance for cells displaying the irrelevant peptide V5-6xHis compared to cells displaying the RBD _318-543_ protein ( **Fig 1F** ). In addition, we found also a higher antibody binding to cells displaying the shorter RBD _462-510_ or the cCD20 _162-191_ peptide in comparison the binding to cells displaying the V5-6xHis peptide with the respective pre-adsorbed sera ( **Fig. 1G -H** ). Taken together, these results strongly indicate that that the elimination of antibodies induced by irrelevant antigens through pre-adsorption of sera paves the way for a subsequent application of a cell-based ELISA, facilitating the specific detection of induced antibodies binding to epitopes on the antigen presented by the tANCHOR display system. The pre-adsorption should be done on the same cell that is subsequently used in the cell-based ELISA measuring antibody binding to a specific target. Only the displayed peptide in the tANCHOR should be exchanged with a non-relevant sequence. Effectively minimizing non-specific antibody binding to cells is essential for cell-based ELISA. Our blocking condition involves 3% bovine serum albumin (BSA), 2% chicken egg albumin (CEA), and goat normal serum. Applying this albumin mixture, along with serum, significantly diminishes background binding, ensuring precise and reliable results, as demonstrated previously (Ivanusic et al., 2016; Ivanusic et al., 2014) . Nevertheless, it is crucial to address a specific limitation in the current experimental design. The assessment in a cell-based assay using human cells is restricted to antibodies induced that do not react with antigens naturally present in human cells. For instance, if the whole-cell immunization involves a displayed human sequence originating from a surface protein, the pre-adsorption step will also remove induced antibodies targeting for that particular antigen. This underlined that the developed technique is most suitable for monitoring serum immunization against bacterial or viral proteins, as well as xenogeneic protein sequences. Additionally, it is important to check for transfection efficiency; otherwise, a low display of antigen on the cell surface will result in reduced binding values.

In summary, through the incorporation of a preincubation step, we demonstrate a novel method for testing serum on the same cells, thereby introducing a new application for testing serum derived from whole-cell immunization.

**Declaration of Interests**

The non-profit organization Peter und Traudl Engelhorn Foundation holds a patent application for the tANCHOR system where D.I. is listed as an inventor. J.M. is a former employee at the company ATG:biosynthetics GmbH (founder H.B.). A.S. and K.H. are associated with the company new/era/mabs GmbH, which provides a proprietary technology platform to generate customized antibodies.

**Author Contribution Statement**

H.B. generated gene synthesis fragments for cloning, and J.M. was involved in SARS-CoV-2 RBD sequence design. D.I. designed the experiments, performed the experiments, analyzed the data, and conceived the original idea. A.S. conducted the immunization study and prepared serum for testing. The manuscript was written by D.I., reviewed and edited by A.S., H.B., N.B., K.H., J.M., and D.I. All authors read and approved the manuscript.

## Methods

**Expression constructs for displaying the antigens Wuhan-Hu-1 RBD _318-543_ , Wuhan-Hu-1 RBD _462-510_ , and cCD20 _162-191_**

The expression constructs ptANCHOR-CD82-RBD _462-510_ -mCherry and ptANCHOR-CD82-cCD20 _162-191_ -mCherry were described in (Bernauer et al., 2023) , ptANCHOR-CD82-RBD _318-543_ -m Cherry described in (Bernauer et al., 2024; Ivanusic et al., 2024) , pCMV-CD82-V5-6xHis-mCherry was described in (Ivanusic et al., 2021) .

**Analysis of protein localization by confocal laser microscopy (CLSM)**

HeLa cells (1 × 10 ^4^ /well) were seeded in a 8 well µ-slide (Ibidi, Martinsried, Germany), and after 24 h transfected using Metafectene reagent with the plasmids ptANCHOR-CD82-RBD _462-510_ -mCherry, ptANCHOR-CD82-cCD20 _162-191_ -mCherry, ptANCHOR-CD82-RBD _318-543_ -m Cherry in the same way as was previously described (Ivanusic and Denner, 2023) . After 24 h post-transfection, the supernatant was removed and replaced with 200 µL of 2% paraformaldehyde (PFA) in 1x PBS and incubated for 20 min. Cells were then washed one time and left in 200 µL of 1xPBS and 1 µL of Hoechst 33342 (ImmunoChemistry Technologies, CA, USA). Protein localization was monitored by confocal laser scanning microscopy employing a Zeiss ZEN (Zeiss, Oberkochen, Germany) smart setup for mCherry (excitation 594 nm/emission 648 nm) and Hoechst 33342 (excitation 405 nm/emission 464 nm).

**Mice serum immunized against the displayed antigens** **Wuhan-Hu-1 RBD _318-543_ , Wuhan-Hu-1 RBD _462-510_ , and cCD20 _162-191_**

The immunization study was conducted following national and international guidelines and approved by the Ministry of Environment, Health, and Consumer Production of the State of Brandenburg, Germany (reference number V3-2347-A16-4-2012). The details are reported in (Bernauer et al., 2023) . Serum for testing in the cell-based ELISA was derived from an immunization study (Bernauer et al., 2023) . Serum was diluted in blocking buffer 1x PBS containing 3% bovine serum albumin (BSA) fraction V (Carl Roth, Karlsruhe, Germany), 2% chicken egg albumin (CEA) (Sigma-Aldrich, Steinheim, Germany), and 10% normal goat serum (Biowest, Nuaillé, France).

**Cell-based ELISA**

HeLa cells (1.5 × 10 ^4^ /well) were seeded in a 96-cell culture well plate (TPP, Trasadingen, Switzerland). After 24 h, cells were transfected with the indicated plasmids, then incubated for an additional 24 h. Fresh medium containing 10% fetal bovine serum was added, and the cells were further incubated for 24 h. They were then washed once. Following this, cells were fixed with 100 µL of 2% PFA for 20 minutes and washed three times with 1xPBS. Subsequently, cells were blocked. At this stage, the 96-well plates are ready for testing the antibody binding to the displayed antigen. All necessary steps performing cell-based ELISA are explained in detail like previously described (Bernauer et al., 2024; Ivanusic et al., 2024) .

**Pre-adsorption of mice serum**

150 µL of diluted mice serum (BALB/c) in blocking buffer, derived from the immunization study was pre-incubated three times for 20 min on HeLa cells transfected with the plasmid DNA pCMV-CD82-V5-6xHis-mCherry before diluted serum was incubated for 1 h on HeLa cells with target specific transfection. Bound IgG on HeLa cells was detected with an rabbit anti-mouse-IgG-HRP antibody (P0260, 1.3 mg/mL, Dako, Agilent Technologies, Denmark) at a dilution of 1:10,000. HRP-bound antibodies were detected with 80 µL TMB solution (Bio-Rad, Munich, Germany). The HRP enzyme reaction was stopped with 100 µL of 2 M H _2_ SO _4_ . Absorbance was measured at a wavelength 450 nm with the reference at 620 nm (450 nm–620 nm) using a TECAN spectrophotometer Infinite 200 (Tecan, Crailsheim, Germany). All further steps including washing and incubation instructions are described in previously work (Ivanusic et al., 2024)

## References

[R1] Balouchi-Anaraki S, Ahmadvand S, Safaei A, Ghaderi A (2021). 4H12, a Murine Monoclonal Antibody Directed against Myosin Heavy Chain-9 Expressed on Acinar Cell Carcinoma of Pancreas with Potential Therapeutic Application.. Iran Biomed J.

[R2] Bernauer Hubert, Schlör Anja, Maier Josef, Bannert Norbert, Hanack Katja, Ivanusic Daniel (2023). tANCHOR fast and cost-effective cell-based immunization approach with focus on the receptor-binding domain of SARS-CoV-2. Biology Methods and Protocols.

[R3] Bernauer Hubert, Maier Josef, Bannert Norbert, Ivanusic Daniel (2024). tANCHOR cell-based ELISA approach as a surrogate for antigen-coated plates to monitor specific IgG directed to the SARS-CoV-2 receptor-binding domain. Biology Methods and Protocols.

[R4] Chen Q, Qiu S, Li H, Lin C, Luo Y, Ren W, Zou Y, Wang Y, Xia N, Huang C (2018). A novel approach for rapid high-throughput selection of recombinant functional rat monoclonal antibodies.. BMC Immunol.

[R5] Dreyer Anita M, Beauchamp Jeremy, Matile Hugues, Pluschke Gerd (2010). An efficient system to generate monoclonal antibodies against membrane-associated proteins by immunisation with antigen-expressing mammalian cells. BMC Biotechnology.

[R6] Huang Chien-Chiao, Cheng Kai-Wen, Hsieh Yuan-Chin, Lin Wen-Wei, Cheng Chiu-Min, Yuan Shyng-Shiou F., Chen I-Ju, Cheng Yi-An, Lu Yun-Chi, Huang Bo-Cheng, Tung Yi-Ching, Cheng Tian-Lu (2019). Use of syngeneic cells expressing membrane-bound GM-CSF as an adjuvant to induce antibodies against native multi-pass transmembrane protein. Scientific Reports.

[R7] Ivanusic Daniel, Madela Kazimierz, Burghard Heidi, Holland Gudrun, Laue Michael, Bannert Norbert (2021). tANCHOR: a&nbsp;novel mammalian cell surface peptide display system. BioTechniques.

[R8] Holzlöhner P, Hanack K (2017). Generation of Murine Monoclonal Antibodies by Hybridoma Technology.. J Vis Exp.

[R9] Ivanusic Daniel, Eschricht Magdalena, Denner Joachim (2014). Investigation of membrane protein—protein interactions using correlative FRET-PLA. BioTechniques.

[R10] Ivanusic Daniel, Denner Joachim, Bannert Norbert (2016). Correlative Förster Resonance Electron Transfer‐Proximity Ligation Assay (FRET‐PLA) Technique for Studying Interactions Involving Membrane Proteins. Current Protocols in Protein Science.

[R11] Ivanusic Daniel, Maier Josef, Icli Suheda, Falcone Valeria, Bernauer Hubert, Bannert Norbert (2024). tANCHOR-cell-based assay for monitoring of SARS-CoV-2 neutralizing antibodies rapidly adaptive to various receptor-binding domains. iScience.

[R12] Ivanusic D, Denner J (2023). The large extracellular loop is important for recruiting CD63 to exosomes.. MicroPubl Biol.

[R13] Leenaars M, Hendriksen CF (2005). Critical steps in the production of polyclonal and monoclonal antibodies: evaluation and recommendations.. ILAR J.

[R14] Molnár Elek (2019). Cell-Based Enzyme-Linked Immunosorbent Assay (Cell-ELISA) Analysis of Native and Recombinant Glutamate Receptors. Methods in Molecular Biology.

[R15] Pandey Shubhi, Roy Debarati, Shukla Arun K. (2019). Measuring surface expression and endocytosis of GPCRs using whole-cell ELISA. Methods in Cell Biology.

[R16] Phakham T, Boonkrai C, Wongtangprasert T, Audomsun T, Attakitbancha C, Saelao P, Muanwien P, Sooksai S, Hirankarn N, Pisitkun T (2022). Highly efficient hybridoma generation and screening strategy for anti-PD-1 monoclonal antibody development.. Sci Rep.

[R17] Rezaei Marzie, Ghaderi Abbas (2018). Monoclonal Antibody Production Against Vimentin by Whole Cell Immunization in a Mouse Model. Iranian Journal of Biotechnology.

[R18] Rezaei Marzieh, Ghaderi Abbas (2017). Production of a Mouse Monoclonal Antibody Against Mortalin by Whole Cell Immunization. Monoclonal Antibodies in Immunodiagnosis and Immunotherapy.

[R19] Shan Zhifu, Yamasaki Takeshi, Suzuki Akio, Hasebe Rie, Horiuchi Motohiro (2016). Establishment of a simple cell-based ELISA for the direct detection of abnormal isoform of prion protein from prion-infected cells without cell lysis and proteinase K treatment. Prion.

[R20] Versteeg HH, Nijhuis E, van den Brink GR, Evertzen M, Pynaert GN, van Deventer SJ, Coffer PJ, Peppelenbosch MP (2000). A new phosphospecific cell-based ELISA for p42/p44 mitogen-activated protein kinase (MAPK), p38 MAPK, protein kinase B and cAMP-response-element-binding protein.. Biochem J.

